# An *in vitro* CT Comparison of Gutta-Percha Removal with Two Rotary Systems and Hedstrom Files

**Published:** 2013-05-01

**Authors:** Pankaj Yadav, Makonahalli Jaganath Bharath, Chickmagravalli Krishnegowda Sahadev, Praveen Kumar Makonahalli Ramachandra, Yogesh Rao, Ambereen Ali, Shahnawaz Mohamed

**Affiliations:** 1 Department of Conservative Dentistry and Endodontics, Maulana Azad Institute of Dental Sciences, New Delhi, India; 2 Department of Conservative Dentistry and Endodontics, Sri Hasanamba Dental College and Hospital, Vidyanagar, Hassan, Karnataka, India; 3 Department of Prosthodontics, Maharana Pratap College of Dentistry and Research Centre, India

**Keywords:** Computed Tomography, Instrumentation, NiTi Files, Root Canal Retreatment

## Abstract

**Introduction:**

To evaluate the efficacy of NiTi mechanical rotary instrumentation and Hedstrom file for gutta-percha/sealer removal computed tomography (CT) was utilized *in vitro*.

**Materials and Methods:**

Thirty extracted human single rooted teeth, each with a single canal were selected. The samples were decoronated with a double faced diamond disk to have 17-mm root; teeth roots were instrumented with K-files up to master apical file #30 using step back technique. Samples were obturated using cold lateral condensation of gutta-percha and AH Plus root canal sealer. The teeth were then randomly divided into three groups of 10 specimens each. After 2 weeks 3-dimensional images of the roots were obtained by CT and the volume of root filling mass was measured. All the canals were then retreated by either the ProTaper retreatment files, Mtwo retreatment files or Hedstrom files. The canals were irrigated with 2 mL of 2.5% sodium hypochlorite irrigating solution during each change of instrument. The volume of remaining filling materials after the retreatment procedures was assessed by CT. Statistical analysis was performed with one-way ANOVA and Tukey’s post hoc test.

**Results:**

Neither of studied systems completely removed the root filling material. No significant difference was observed between the rotary systems. The volume of remaining filling materials was significantly less in rotary instrumentation than hand files. There was no significant difference for debris extruded from the apical foramen between the groups.

**Conclusion:**

Under the experimental conditions, Mtwo and ProTaper retreatment files left less gutta-percha and sealer than H files; however, complete removal of filling materials was not achieved by the three systems investigated.

## 1. Introduction

Even with the most modern techniques, failures in endodontic treatment are sometimes inevitable and it is often necessary to retreat the root canals (1). Endodontic failure might occur due to persistence of bacteria in the root canal system as a consequence of insufficient cleaning, inadequate obturation or when there is coronal/apical leakage (2). Clinically, failure of endodontic treatment is determined on the basis of radiographic findings and clinical signs or symptoms of the treated teeth (3). The main goal of non-surgical retreatment is to re-establish healthy periapical tissues. The procedure requires the complete removal of the root filling, ingressed bacteria and further refilling (4).

The most commonly used root canal filling material is gutta-percha in combination with a root canal sealer, because the use of gutta-percha without a sealer fails to produce a hermetic seal (5, 6). Therefore, re-treatment of previously filled canals demands that the gutta-percha and the sealer must be removed from the canal walls and anatomical ramifications to ensure complete cleaning of the root canal system during the chemomechanical preparation and application of antibacterial dressings (6). Removing filling material from inadequately prepared or filled root canal systems is necessary because this material causes a mechanical barrier that hinders contact of irrigating solutions and intracanal dressings to the root canal walls (7). Removal of filling material also uncovers remaining necrotic tissue or bacteria that might be responsible for periapical inflammation and thus post treatment disease (4). In addition, apical extrusion of debris also appears to occur with all instrumentation techniques which may cause acute exacerbations of chronic inflammatory conditions. Therefore, practical methods for removing this material from the root canal has been investigated (5). One of the greatest technical difficulties faced by endodontists is achieving complete removal of old filling material (7) which is directly related to canal preparation and filling techniques, the type of sealer used as well as the time elapsed since the original treatment (8).

The removal of gutta-percha using hand files with or without solvent can be a tedious, time-consuming process, especially when the root filling material is well condensed (9). Therefore, it is necessary to identify the optimum file system that will allow most effective removal of obturation material from the root canal (10). Several techniques can be used to remove the gutta-percha, including the use of stainless steel hand files (11, 12) nickel-titanium (NiTi) rotary instruments (13-16), heat-bearing instruments (17, 18), ultrasonic instruments (19-21) and lasers (22, 23). The usage of rotary instruments in the retreatment process presents advantages concerning clinical time reduction (24). Two new NiTi systems have recently been designed for gutta-percha removal. They are the ProTaper Universal retreatment files (Dentsply Maillefer, Ballaigues, Switzerland) and Mtwo retreatment rotary files (Sweden and Martina, Padova, Italy).

ProTaper Universal system comprises 3 flexible instruments (D1, D2 and D3), of which the tapers and tip diameters are equivalent to size 0.09/0.30 mm, 0.08/ 0.25 mm and 0.07/0.20 mm, respectively. These instruments are specially designed for root filling removal from the coronal, middle and apical portions of root canals. Mtwo retreatment system consists of 2 retreatment instruments (size 15, taper 0.05 and size 25 taper 0.05) with cutting tips for efficient removal of gutta-percha fillings. Remaining filling debris has been assessed by conventional radiography, splitting teeth longitudinally or making teeth transparent (25). However, sectioning can disturb the remaining filling materials. Radiographic produced images are two-dimensional representations of three-dimensional structures and may be subject to magnification and distortion (26). Computed tomography (CT) may be a viable alternative for the qualitative and quantitative evaluation of retreatment procedures.

The aim of this in vitro study was to compare ProTaper, Mtwo retreatment and Hedstrom files in gutta-percha removal from root canal walls using CT scan.

## 2. Material and Methods

Forty three human mandibular single rooted premolars extracted for orthodontic reasons were collected. Preoperative mesiodistal and buccolingual radiographs were taken of each root to confirm the canal anatomy, full development of root, absence of root fillings, pins, internal resorptions or localized/diffuse calcifications.

Access preparation was performed using high speed diamond burs with copious water spray. A size #10 K-file was placed in the canal until it was visible at the apical foramen. The teeth which had apical diameter wider than size #15 K-file were excluded. Then the rest of the teeth were verified radiographically for degree of curvatures <10° (Schneider 1971). Finally thirty teeth were selected.

### 2.1. Root Canal Treatment

The working length was determined by subtracting 1mm from afore mentioned measurement. The crowns were removed with a diamond disk to leave 17 mm root. The root canal was prepared using K-files with step back technique. Instrumentation was standardized with a size #30 K-file reaching full working length, a size #55 file 5 mm coronally and final coronal flaring with Gates Glidden. When the instrumentation of root canal was completed, 17% EDTA (ethylene diamine tetra acetic acid) was applied for 3 min in order to remove smear layer and the canals were again irrigated with 2 mL of 2.5% NaOCl followed by final rinse with 5 mL of saline solution.

### 2.2. Canal Obturation

The root canals were dried with paper points and obturated by lateral compaction technique, using gutta-percha cones (Dentsply Maillefer, Baillaigues, Switzerland) and AH Plus sealer (Dentsply DeTrey, Zurich, Switzerland). Coronal access was sealed with a temporary filling material (Cavit G; 3M ESPE, Seefeld, Germany). The specimens were stored at 37ºC in 100% humidity for 2 weeks. Instrumentation of the samples was done by an endodontic post graduate student.

The teeth were then randomly numbered. Before obtaining the initial CT images, the temporary filling material was removed. Using an A4 sheet, spaces corresponding to the roots were demarcated and identified with the number of each root (1 to 30). The roots were fixed on these locations with double side tape and laid out side by side with their buccal surface turned to the left and the outline of teeth was marked with a pencil on double side tape. Three dimensional images of the roots were obtained using the Somatom Sensation cardiac 16 Multi Slice CT (Siemens AG Medical Solutions, Erlangan, Germany). Total of fifteen sections with 1mm distance/thicknesses were cut. After obtaining CT scans from all specimens, the total volume of the root-filling mass in each canal was measured in cm^3^ using the CT scanner proprietary software (Syngo volume software, Siemens Siemens, Erlangen, Germany) by an expert radiologist. Total volume of filling material in cm^3^ was recorded in a spread sheet as calculated by the software.

### 2.3. Retreatment Technique

In each sample, 5-mm of filling material was removed from the cervical part using Gates Glidden burs sizes 2 and 3 at 5000 rpm. Then a drop of xylene solvent (Ajax Finechem Pty. Ltd, India) was introduced into each canal and left to act for 2 min. Two to three additional drops of solvent were required to reach the working length. During retreatment the canals were constantly irrigated with 2 mL 2.5% NaOCl.

All the rotary instruments were used at a constant speed of 300 rpm and torque recommended by the manufacturers. Rotary instrumentation of the ProTaper and Mtwo retreatment files was performed using a 16:1 reduction gear handpiece with an electric motor (X-Smart; Dentsply, Baillaigues, Switzerland). All teeth were treated by the post graduate students.

#### 2.3.1. Group I: ProTaper retreatment system

All the 3 ProTaper Universal System retreatment files were used sequentially with crown down technique, until the working length was reached using a brushing action with lateral pressing movements. The D1 ProTaper file was used to remove the filling material from the cervical third of the root canal. A D2 ProTaper file was used in the coronal two thirds of the root canal. The D3 ProTaper file was used with light apical pressure until the working length was reached and no further filling material could be removed.

#### 2.3.2. Group II: Mtwo retreatment system

The Mtwo retreatment file was used according to the manufacturer’s instructions. Retreatment was initiated by placing tip of R2 size 25, 0.05 taper retreatment file on the gutta-percha. The canals were instrumented to the working length using Mtwo R2 file with circumferential filing and a lateral pressing movement. Progression of the rotary files was performed by applying slight apical pressure and frequently removing the files to inspect the blade and clean the debris from the flutes.

#### 2.3.3. Group III: Hedstrom files

Hedstrom file (H type; Dentsply, Maillefer, Ballaigues, Switzerland) sizes 45, 40, 35, 30 and 25 were used in crown down manner using circumferential quarter turn push-pull filing motion to remove gutta-percha and sealer from the canal until the working length was reached with a size 25 Hedstrom file. Sizes 15 and 20 Hedstrom files were used to gain deep penetration down the canal until working length was reached. A step back procedure was performed in 1mm increments to size 55 file. On withdrawal, the files were cleansed of any obturating material before being re-introduced into the root canal. Each file was discarded after instrumentation of five canals. Any deformed file was discarded. Retreatment was considered complete when no filling material was observed on the file and the canal walls were smooth and free of visible debris.

### 2.4. Evaluation methods

For all teeth, three types of data were recorded:

#### 2.4.1. Volume of remaining filling materials using CT

**Figure 1.**
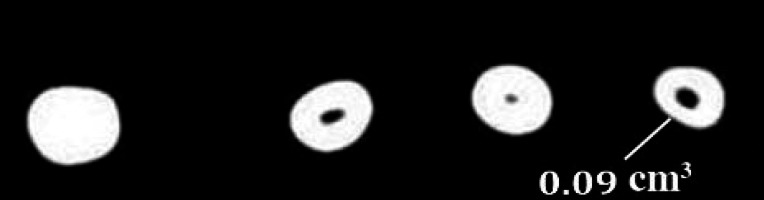
The mean volume of remaining filling materials after removal of obturation material using Hedstrom files

**Figure 2.**
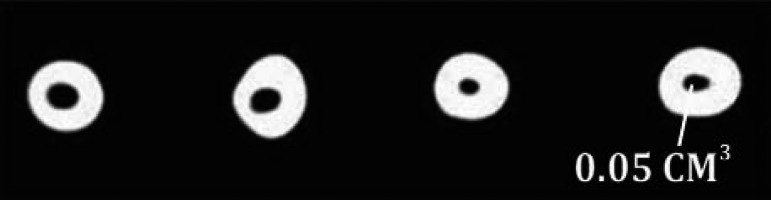
The mean volume of remnant filling materials following removal of obturants with ProTaper retreatment files

**Figure 3.**
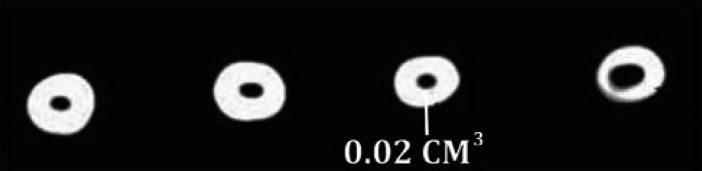
The mean volume of remaining filling materials after retreatment with Mtwo retreatment files

After root filling removal, the specimens were placed back on the demarcated A4 paper sheet and fixed in the same positions as for the initial CT scanning. Another CT scan of each root was taken. The volume of remaining filling materials inside the canal was calculated (Figures 1, 2 and 3) in cm^3^ and recorded on a spreadsheet as final volume. The volume was analyzed using the same software and by the same radiologist, who was blind to the three investigated groups.

#### 2.4.2. Apically extruded debris

The amount of apically extruded debris were detected visually during the filling removal was evaluated by the operator with the following scoring system: 0, no extruded debris or no filling material escaping through the foramen; 1, minimal extruded debris or small amounts of filling material escaping through the foramen; 2, moderate extruded debris or greater amounts of filling material escaping through the foramen; and 3, severe extruded debris or even greater amounts of filling material escaping through the foramen.

#### 2.4.3. Procedural errors

The number of instruments that fractured was also recorded. Statistical Analysis was performed using one-way ANOVA and Tukey’s post hoc multiple range tests.

## 3. Results

All retreatment techniques used in this study left some filling material inside the root canal. The mean volume of remaining filling materials (mean) in the canal were less with the Mtwo retreatment (0.011 cm^3^) and ProTaper rotary retreatment systems (0.012 cm^3^) compared to hand instruments (0.024 cm^3^) and the difference was statistically significant. There was no significant difference between rotary systems. Mtwo retreatment system showed the least volume of remaining gutta-percha/sealer among the three groups (Tables 1 and 2). Evaluating the debris extruded from the apical foramen showed that there was no significant difference among the groups. Overall, there were two fractured files in the Hedstrom group.

**Table 1.** Volume of remnant filling material

Groups	Files	Mean Volume Remaining (cm^3^)	Standard Deviation (cm^3^)
**1**	**ProTaper Retreatment File**	0.012	100±2.52
**2**	**Mtwo Retreatment File**	0.011	88.84±1.62
**3**	**H File**	0.024	77.09±2.89

K-W ANOVA, H=6.05, P<0.05 Significant

**Table 2.** The results of Mann-Whitney U test of remnant filling material

		
**Groupwise Comparisons (*P*-values)**	1 vs 2	0.73 Non-significant
	1 vs 3	0.05 Significant
	2 vs 3	0.10 Non-significant

## 4. Discussion

Nonsurgical endodontic retreatment is an attempt to re-establish healthy periapical tissues after inefficient treatment or reinfection of an obturated root canal system (14). This procedure can uncover residual necrotic tissues or bacteria that may be responsible for persistent periapical inflammation and allow further cleaning and refilling of the root canal system (25). The success of non surgical endodontic retreatment depends on the effective elimination of necrotic tissue, bacteria and infected obturation material such as gutta-percha and cements from root canals (27). Within the limitations of the present study, the results showed that all files helped in removal of obturation material; however, there were some filling material remained in root canal. Overall NiTi rotary instruments were more effective than stainless steel H-files in the removal of root filling material.

In this study, the teeth were decoronated to allow specimen standardization by eliminating some variables, such as dental crown anatomy and the root canal access (11). Rotary and hand files were used for cleaning of root-filled canals because they are commonly used in endodontic retreatment. Root canals were filled with AH Plus, which is a resin-based sealer of strong sealing ability due to great adhesion to dentin and consequent reduced bacterial leakage (28).

Several methodological approaches have been popular in endodontic retreatment research. The currently used methods include longitudinal cleavage of the teeth, association of longitudinal and transversal cleavage for evaluation of the cervical, middle and apical thirds separately (29). In addition, computed tomography and operating microscopes have been employed for this purpose (30). However, longitudinal cleavage of teeth may result in displacement of the filling debris to be evaluated and compromise the accuracy of the measurements (29). Radiographic images produced are two-dimensional representations of three-dimensional structures and may be subject to magnification and distortion; also fine layers of debris may not be sufficiently radiopaque to be picked up (26). We used CT scanning to outweigh the limitations of the methodologies previously applied in endodontic retreatment studies. This non-invasive method allows visualization of morphological features in detail (31). Use of computed tomography is an acceptable method for retreatment research but its use for clinical retreatment is not recommended because of large radiation exposure (32). Among all the methods, computed tomography appears to be simple, efficient and sensitive enough to identify small area of residual gutta-percha/sealer on the canal walls. CT scanning offers three-dimensional reproducible data and allows the assessment of endodontic retreatment by comparing the amount of debris inside the root canals before and after removal procedures (29).

We had no broken rotary instruments, probably due to the utilization of low-torque motor with constant speed. In addition, each set of instruments only prepared five root canals and the application of solvent xylene also helped to prevent iatrogenic errors like broken instruments.

Efficacy of the ProTaper Universal rotary retreatment system for gutta-percha removal from root canals was evaluated by Gu et al. It was concluded that all techniques left gutta-percha/sealer remnants on root canal walls. The ProTaper Universal rotary retreatment system removed gutta-percha more efficiently compared with other traditional techniques with Hedstrom files and K-files. The better performance of ProTaper Universal retreatment instruments was attributed to the three progressive tapers and length design of D1, D2 and D3 files (25). Our study concurs with Gu et al.

Efficacy of two rotary NiTi instruments in the removal of gutta-percha during root canal retreatment in comparison with hand Hedstrom files was evaluated by Saad et al. It was concluded that ProTaper and K3 required significantly less time for filling material removal than hand instruments. This finding could probably be due to the inherent characteristic design of ProTaper and K3 rotary files (33). Our study agrees with study by Saad et al.

Effectiveness of the Mtwo R, ProTaper retreatment files and a Hedstrom manual technique in the removing gutta-percha, Resilon and EndoRez was compared by Somma et al. It was found that all instruments left remnants of all the filling material and debris on the root canal walls irrespective of the root filling material used. Both the engine-driven NiTi rotary systems proved to be safe and fast devices for the removal of endodontic filling material (34); confirmed by our study.

Efficacy of the R-Endo rotary NiTI instrumentation system and hand instrumentation to remove gutta-percha or Resilon from root canals was evaluated by Fenoul et al. It was concluded that time to reach the working length and for removal of filling was lower with R-Endo than with Hedstrom files. This is probably due to instrument design of R-Endo (35); the results of present study confirm these findings. Overall NiTi rotary instruments were more effective than stainless steel H-files in the removal of root filling material.

In this study, Mtwo retreatment files left less gutta-percha followed by ProTaper retreatment files and Hedstrom files. Among all the systems, better performance of Mtwo retreatment files is attributed to the design of the instrument. The Mtwo retreatment files have an S-shaped cross-section, an increasing pitch length in the apical-coronal direction and a cutting tip. Therefore, these instruments are characterized by a positive rake angle with two cutting edges. The cutting blades form long, vertical spirals ensuring better control of instrument progression through the canal. As they have sharp blades, it is possible to cut through the canal and reach the apical end-point whilst by passing obturation material. Also, unlike some of the NiTi instruments, Mtwo rotary instruments do not require a crown-down instrumentation sequence.

## 5. Conclusion

The rotary retreatment and hand files used in this in vitro study left some filling materials inside the root canal. The rotary NiTi instruments were significantly more effective than Hedstrom files in removing gutta-percha during retreatment but there was no significant difference between the two rotary instruments with CT radiography. Computed tomography can be considered a good technology for retreatment research.
